# Supplementary material for: Determinants of Changes in the Diet Quality of Japanese Adults during the Coronavirus Disease 2019 Pandemic
Source: Nutrients. 2022 Dec 27;15(1):131. doi: 10.3390/nu15010131 (PMC9823767; doi:10.3390/nu15010131)
Supplement: Supplementary file 1 [file nutrients-15-00131-s001.zip › nutrients-2099173-supplementary.pdf]

Table S1. Food group intakes according to changes in the quality of dietary patterns

	Unadjusted							Adjusted						
	IDQ (*n* = 214)		WDQ (*n* = 233)		Others (*n* = 1,654)		*P*[*1]	IDQ (*n* = 214)		WDQ (*n* = 233)		Others (*n* = 1,654)		*P*[*2]
	**Mean**	**SD**	**Mean**	**SD**	**Mean**	**SD**		**LSM**	**SE**	**LSM**	**SE**	**LSM**	**SE**	
**Food groups for which daily consumption is recommended**[*3]														
Whole grains	1.94	2.25[a]	1.48	1.93	1.58	2.04[b]	0.033	1.91	0.14	1.46	0.13	1.59	0.05	0.049
Fish and shellfish (excluding processed products)	2.32	1.66[a]	1.84	1.34[b]	2.00	1.50[b]	0.002	2.31	0.10[a]	1.85	0.10[b]	2.00	0.04[b]	0.004
Lean meats (excluding processed products)	2.52	1.83[a]	2.02	1.59[b]	1.98	1.55[b]	<0.001	2.47	0.11[a]	2.01	0.10[b]	1.99	0.04[b]	<0.001
Eggs	4.16	2.10[a]	3.35	2.19[b]	3.43	2.18[b]	<0.001	4.13	0.15[a]	3.33	0.14[b]	3.44	0.05[b]	<0.001
Milk and dairy products (unsweetened)	4.22	2.47[a]	3.47	2.49[b]	3.72	2.52[b]	0.005	4.18	0.17[a]	3.44	0.16[b]	3.73	0.06[b]	0.005
Soy and soy products	4.02	2.30[a]	2.75	2.02[b]	3.24	2.24[c]	<0.001	4.00	0.15[a]	2.75	0.14[b]	3.25	0.05[c]	<0.001
Green and yellow vegetables	4.65	2.03[a]	3.35	2.31[b]	3.80	2.28[c]	<0.001	4.58	0.15[a]	3.33	0.14[b]	3.81	0.05[c]	<0.001
Other vegetables	5.08	1.86[a]	3.60	2.34[b]	4.08	2.26[c]	<0.001	5.03	0.15[a]	3.57	0.14[b]	4.09	0.05[c]	<0.001
Seaweeds	2.72	2.10[a]	1.82	1.66[b]	2.08	1.89[b]	<0.001	2.72	0.13[a]	1.83	0.12[b]	2.08	0.05[b]	<0.001
Mushrooms	2.65	2.02[a]	2.07	1.74[b]	2.12	1.79[b]	<0.001	2.60	0.12[a]	2.06	0.12[b]	2.13	0.04[b]	<0.001
Potatoes	1.93	1.52[a]	1.47	1.14[b]	1.60	1.30[b]	<0.001	1.89	0.09[a]	1.47	0.09[b]	1.60	0.03[b]	0.002
Fruits (excluding processed products)	3.23	2.35[a]	2.28	2.19[b]	2.35	2.19[b]	<0.001	3.18	0.15[a]	2.25	0.14[b]	2.36	0.05[b]	<0.001
**Food groups for which daily consumption is not recommended**														
Processed meat or fish products	2.04	1.65	2.26	1.71	2.05	1.59	0.155	2.03	0.11	2.27	0.11	2.05	0.04	0.152
Snacks and desserts	3.06	2.30[a]	3.77	2.24[b]	3.03	2.25[a]	<0.001	2.97	0.15[a]	3.73	0.15[b]	3.05	0.06[a]	<0.001
Alcoholic beverages	2.02	2.22	2.32	2.33	2.14	2.32	0.365	2.21	0.15	2.38	0.14	2.10	0.05	0.173
Sweetened beverages	2.16	2.25	2.45	2.39	2.11	2.21	0.089	2.21	0.15	2.46	0.15	2.10	0.06	0.067
Frozen meals	1.34	1.10[a]	1.91	1.50[b]	1.55	1.37[a]	<0.001	1.35	0.09[a]	1.93	0.09[b]	1.55	0.03[a]	<0.001
Instant products	1.19	1.04[a]	1.89	1.60[b]	1.41	1.25[c]	<0.001	1.23	0.09[a]	1.91	0.08[b]	1.41	0.03[a]	<0.001

*1 p values were calculated using ANOVA. Multiple comparison among groups were based on Bonferroni. There are significant differences between different alphabets. *2 p values were calculated using ANCOVA. Adjusted models include gender, age groups, household income change, and household economic status before the COVID-19. There are significant differences between different alphabets. *3 The average score was calculated by scoring 6.5 points for "almost every day", 3.5 points for "once every two days", 1.5 points for "1–2 times a week", and 0.5 points for "almost never eat".
SD: standard deviation, LSM: least squares mean, SE: standard error.
